# Standard versus short stem cemented Exeter^®^ when used for primary total hip arthroplasty: a survivorship analysis

**DOI:** 10.1186/s42836-023-00200-8

**Published:** 2023-09-03

**Authors:** Nick D. Clement, Liam Z. Yapp, Leo D. Baxendale-Smith, Deborah MacDonald, Colin R. Howie, Paul Gaston

**Affiliations:** 1 Edinburgh Orthopaedics, Royal Infirmary of Edinburgh, Little France, Edinburgh, ED16 4SA UK; 2 Department of Orthopaedics, University of Edinburgh, Little France, Edinburgh, EH16 4SB UK

**Keywords:** Total hip arthroplasty, Cemented, Stem, Length, Short, Outcome, Survival

## Abstract

**Aims:**

The aims were to compare the survival of the cemented standard (150 mm) with the short (DDH [35.5 mm offset or less], number 1 short stem [125 mm options of 37.5 mm, 44 mm, 50 mm offset] and revision [44/00/125]) Exeter^**®**^ V40 femoral stems when used for primary total hip arthroplasty (THA).

**Methods:**

Patients were retrospectively identified from an arthroplasty database. A total of 664 short stem Exeter^**®**^ variants were identified, of which 229 were DDH stems, 208 number 1 stems and 227 revision stems were implanted between 2011 and 2020. A control group of 698 standard Exeter^**®**^ stems used for THA was set up, and were followed up for a minimum of 10 years follow-up (implanted 2011). All-cause survival was assessed for THA and for the stem only. Adjusted analysis was undertaken for age, sex and ASA grade.

**Results:**

The median survival time for the short stems varied according to design: DDH had a survival time of 6.7 years, number 1 stems 4.1 years, and revision stems 7.2 years. Subjects in the short stem group (*n* = 664) were significantly younger (mean difference 5.1, *P* < 0.001) and were more likely to be female (odds ratio 1.89, 95% CI 1.50 to 2.39, *P* < 0.001), compared to the standard group. There were no differences in THA (*P* = 0.26) or stem (*P* = 0.35) survival at 5 years (adjusted THA: 98.3% vs. 97.2%; stem 98.7% vs. 97.8%) or 10 years (adjusted THA 97.0% vs. 96.0 %; stem 96.7% vs. 96.2%) between standard and short stem groups, respectively. At 5 years no differences were found in THA (DDH: 96.7%, number 1 97.5%, revision 97.3%, standard 98.6%) or stem (DDH: 97.6%, number 1 99.0%, revision 97.3%, standard 98.2%) survival between/among the different short stems or when compared to the standard group.

**Conclusion:**

The Exeter^**®**^ short stems offer equivocal survival when compared to the standard stem at 5- to 10-year follow-up, which does not seem to be influenced by the short stem design.

## Introduction

**Fig. 1:**
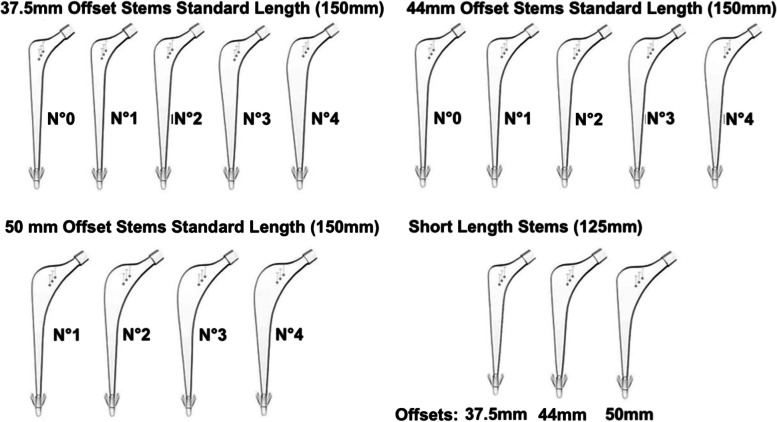
This figure illustrated some of the available Exeter^**®**^ stem options in relation to the standard length (150 mm) and the newly available number 1 short stem (125 mm) options that are available in 37.5 mm offset and greater. The number (No) is related to the thickness of the stem, with a greater number indicating a thicker stem

The polished cemented Exeter^**®**^ stem is an established implant that was designed in 1970 and has an associated excellent long-term survivorship when used for total hip arthroplasty (THA) [1]. The original length of the Exeter^**®**^ stem was 150 mm, however, shorter stem options, with time, have become readily available to aid restoration of patients’ native anatomy. The 35.5 mm offset stem comes in 125 mm as standard. In 2004, a revision stem designed for cement-in-cement revision was made available, with an offset of 44 mm and a length of 125 mm [2]. This short revision stem (SRS) is slimmer both distally and front to back compared to standard stems. More recently, the stems with an offset of 37.5 mm or more have been available with a short stem option of 125 mm, which is only available in number 1 (Fig. 1), and obtained a CE mark in 2014 and are in routine usage in the UK since 2015 for primary THA. The aim of the shorter cemented stem was to facilitate implantation into patients with smaller proximal femoral canals (Dorr type A [3]), to enable retention of bone stock, that would have otherwise required removal of cortical bone to retain an adequate cement mantle if a standard stem was employed.

Cementless short stem implants are well established and have been used since 1989 and are thought to aid physiological loading and preserve bone stock [4], with equivocal survival and clinical outcomes [5]. However, this may not be the same for a cemented short stem, which is associated with an increased incidence of varus malalignment that may result in inferior implant survival with stem loosening [6–8]. The Exeter^**®**^ SRS (44/00/125) has been successfully employed for revision cases [2] but has also been used off-label for primary THA, which is associated with a higher rate of revision [9, 10]. Wyatt et al. [11] demonstrated a greater revision risk associated with the Exeter^**®**^ short stem with an offset of less than 37.5 mm for THA, but no difference was observed for short stems of 37.5 mm or more at a maximum of 3 years follow-up, using data from the New Zealand Arthroplasty registry. Martin et al. [12] reported a case series of 60 patients undergoing THA with an Exeter^**®**^ short stem of 37.5mm or more at a maximum of 3 years follow-up and reported no revisions of the stem. Therefore, there are limited data reporting the survival of the Exeter^**®**^ short stem, especially the recent short stem number 1 implants with a 37.5 mm or more offset, when used as part of a THA.

The primary aim of this study was to compare the survival of the cemented standard (150 mm) with the short (125 mm) Exeter^**®**^ V40 stems when used for primary THA. The secondary aims were to compare the survival of the different short stems that were available (developmental dysplasia of the hip (DDH) stem [35.5 or less], number 1 short stem [offset options of 37.5, 44, 50 mm] and SRS [44/00/125]) and the demographic differences among these groups.

## Methods

Ethical approval was obtained from the regional ethics committee (Research Ethics Committee, South-East Scotland Research Ethics Service, Scotland [11/AL/0079, 16/SS/0026]) for the arthroplasty database used in this study. The data collection was carried out in accordance with the GMC guidelines for good clinical practice and the Declaration of Helsinki.

A case-control study was undertaken. Participants were retrospectively identified from an arthroplasty registry held at the study center. From 2011 all implant bar codes were scanned into the arthroplasty registery with patient demographics, and the implant-specific codes were used to identify Exeter^**®**^ stems retrospectively. The implant codes were obtained from the Global Unique Device Identification Database (GUDID). GUDID is an open-access database that contains key medical device information such as unique device identifiers (UDI), which are submitted to the United States Food and Drug Administration (FDA) [13]. In addition, this was crossed checked with the patient’s notes to ensure they had a primary THA. The same database also records the ASA grade of the patient. Patients undergoing a primary THA for osteoarthritis during the year 2011 using a standard Exeter^**®**^ stem (150 mm) were designated the control group. The short stem group included patients undergoing a primary THA between 2011 and 2020 (for a minimum of 2-year follow-up) using an Exeter^**®**^ stem of less than 150 mm. The short stem group was further divided into three sub-groups: DDH stems, number 1 short stem and SRS groups. The DDH group included 35.5 mm offset stems or smaller (Asian specific), which was available for the entire time period assessed. The number 1 short stem group included stems (offsets 37.5, 44, 50) that were available in standard 150 mm length but are also available in short length (125 mm) from 2015 to 2020. The SRS was defined as the 44mm offset double zero stems which were originally designed for cement in cement revision was available for the entire time period assessed.

Patients received either an “original” (150 mm) or “short” cemented Exeter^**®**^ stem as part of their routine THA at the study center. The short stem was employed, over the standard stem, when the proximal femur would not allow a standard broach to be inserted into the planned position due to a narrow canal. Rather than removing distal cortical bone from the canal, a short stem was then used, retaining cortical bone stock and maintaining a 2-millimeter cement mantle around the implant. A standard operative technique was employed by all surgeons, using an anterolateral or posterior approach, and Exeter^**®**^ femoral component and Contemporary^TM^ Flanged acetabular component (Stryker), Low Profile (Stryker), Trident^TM^ (Stryker) or Restoration^TM^ ADM (Stryker). The routine postoperative patient care protocol was used. Venous thromboembolism prophylaxis was left at the surgeon’s discretion and relative risk to the patient, however during this period aspirin was most commonly administered [14].

Survival of the THA and the stem were specifically assessed. The patient’s notes and the National Picture Archiving Communication System for Scotland (Kodak CareStream) were assessed. Patients undergoing revision were identified and classified as either related to the THA as a whole or related to the stem specifically (aseptic loosening, periprosthetic fracture, implant fracture). Revision for infection and recurrent dislocation was assigned a THA failure rather than specifically for the stem. Patient mortality was obtained from the patient’s medical notes. If the patient was not recorded as deceased and had not been revised, it was assumed that they were alive with a functioning prosthesis. The study center is the only National Health Service health board for the catchment population. Survival was assessed over the period of the patient’s lifetime or to the end of their follow-up.

### Statistical analysis

Statistical analysis and data handling were performed by using R Studio (Version 1.3.959) (Boston, Massachusetts, USA). Depending on the distribution of data, parametric or non-parametric tests were used to assess continuous variables for significant differences between groups. Dichotomous variables were assessed using a Chi-square test for between-group comparisons or a Fisher’s exact test if one of the cell types was less than five.

Survivorship was examined using Kaplan-Meier estimates with 95% confidence intervals. The event of interest was revision, and patients were censored for death or reaching the end of the follow-up period. We considered a revision to any part of the THA construct (all-cause) or femur-only. Differences between stem types were assessed using the log-rank method. Adjusted Kaplan-Meier survival curves were created by performing reweighting. Reweighting was performed by using the cohort’s distribution of the variables age at surgery, sex and ASA as the target (empirical) distribution. For all analyses, a *P*-value of <0.05 was defined as statistically significant.

A post hoc power calculation using G-Power 3.1 [15] demonstrated a power of 83.0% for the cohort used (standard *n* = 698 versus short stem* n* = 665) to demonstrate a 2.2% difference in survival, based on that previously observed for the SRS compared to the standard stem [10], using a one-tailed analysis (assumed short stem was associated with a worse/lower survival) and an alpha of 0.05.

## Results

A total of 664 short stem Exeter^**®**^ variants were identified, of which 229 (34.5%) were DDH stems, 208 (31.3%) short stems and 227 (34.2%) SRS, with an overall median survival time of 5.2 years (interquartile range [IQR] 3.6 to 8.0). The control group consisted of 698 standard Exeter^**®**^ stems, with a median survival time of 10.8 years (IQR 10.2 to 11.1). The median survival time for the short stems varied with design (Table 1), with a shorter time for the number 1 short stem group (*n* = 208) due to the fact that they were only available from 2015. Overall, subjects in the short stem group (*n* = 664) were significantly younger (Short Stem 62.2 (IQR 51.6–71.6) vs. Standard 68.0 (59.0–75.0), mean difference -5.1, 95% CI -6.5 to -3.7, *P* < 0.001, Mann-Whitney U test) and were more likely to be female (odds ratio [OR] 1.89, 95% CI 1.50 to 2.39, *P* < 0.001, Chi-Square) compared to the standard group. However, more specifically, subjects in the DDH stem group were predominately female (*n* = 219, 95.6%) and were significantly younger than those in the short stem (*n* = 101, 48.6%) and SRS (*n* = 178, 78.4%) groups (Table 1, *P* < 0.001). In contrast, subjects in the number 1 short stem group were predominantly male (*n* = 108, 51.4%), significantly greater/older than in DDH (OR 23.20, 95% CI 11.64 to 46.24, *P* < 0.001) and SRS (OR 3.85, 95% CI 2.54 to 5.84, *P* < 0.001) groups. There was no significant difference in the ASA grade (*P* = 0.852)

**Table 1:** Survival time, patient demographics and ASA grade for standard and short stem groups

**Variables**	**Standard Exeter, *n* = 698**	**DDH Exeter, *n* = 229**	**No.1 Short Stem Exeter, *n* = 208**	**Short Revision Stem, *n* = 227**
Survival time				
Median (IQR)	10.8 years (10.2–11.1)	6.7 years (4.0–8.4)	4.1 years (3.3–4.8)	7.2 years (4.6–9.1)
Age				
Median (IQR)	68 (59–75)	59 (51–72)	62 (52–71)	63 (54–72)
Sex				
Male	270 (38.7%)	10 (4.4%)	107 (51.4%)	49 (21.6%)
Female	428 (61.3%)	219 (95.6%)	101 (48.6%)	178 (78.4%)
ASA Grade				
1	102 (14.6%)	30 (13.1%)	31 (14.9%)	35 (15.4%)
2	437 (62.6%)	153 (66.8%)	138 (66.3%)	148 (65.2%)
3	156 (22.4%)	44 (19.2%)	39 (18.8%)	43 (18.9%)
4	3 (0.4%)	2 (0.9%)	0 (0%)	1 (0.5%)

*IQR* Interquartile range, *DDH* Developmental dysplasia of hip, *ASA* American Society of Anaesthesiologists

**Fig. 2:**
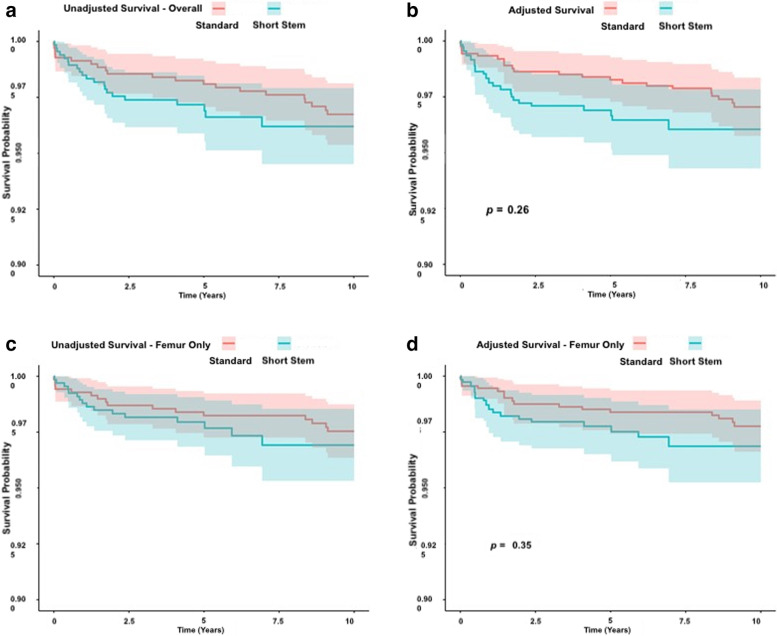
Kaplan Meier survival curves for unadjusted THA (**A**) and stem only (**B**) survival, and THA (**C**) and stem only (**D**) survival adjusted for age, sex and ASA grade

**Fig. 3:**
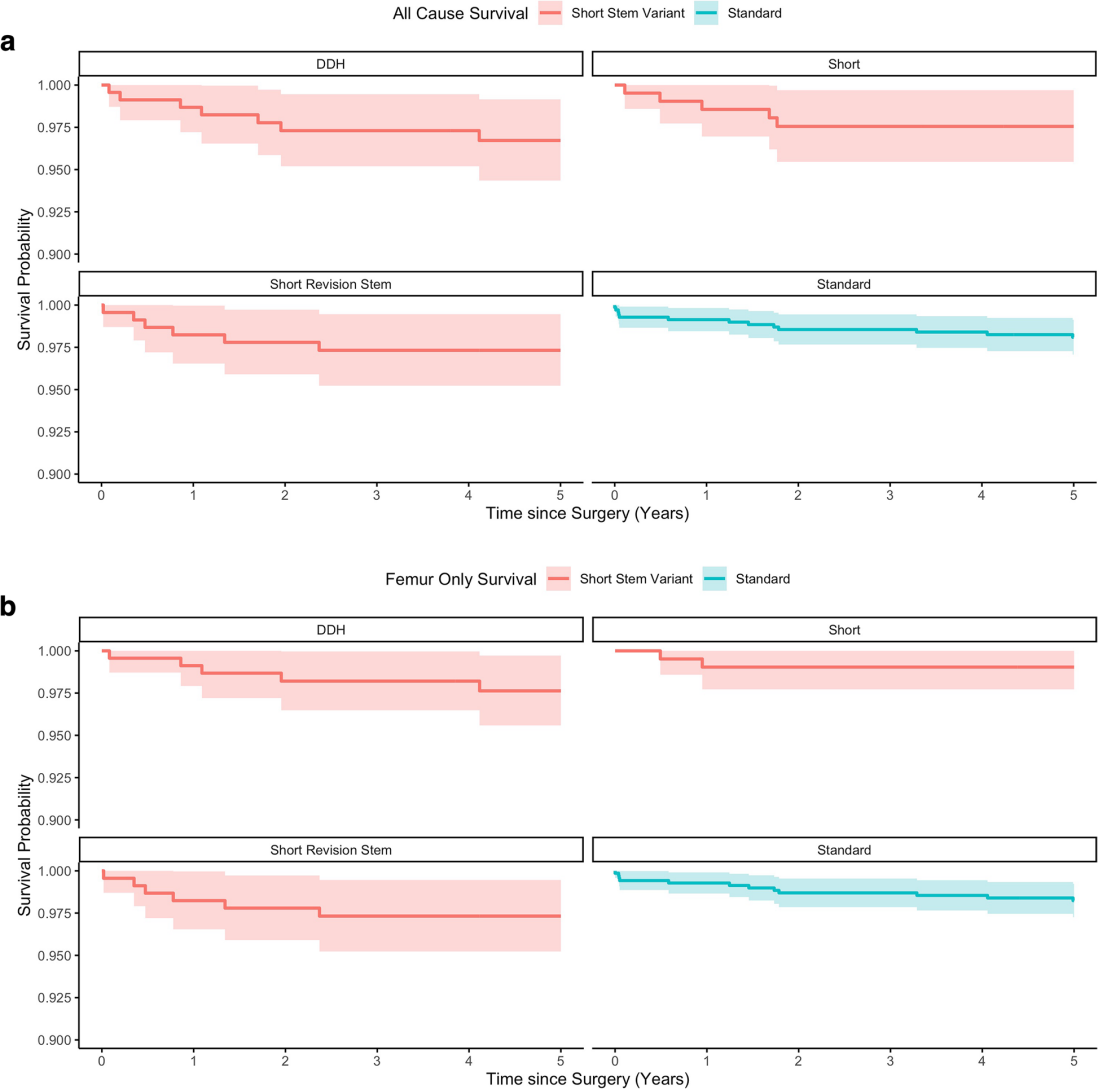
Kaplan Meier survival curves for THA (**A**) and stem only (**B**) survival in terms of standard and short stem group

No differences were revealed in THA (*P* = 0.26) or stem-only (*P* = 0.35) survival at 5 years (adjusted THA: 98.3% vs. 97.2%; stem 98.7% vs. 97.8%) or 10 years (adjusted THA 97.0% vs. 96.0%; stem 96.7% vs. 96.2%) between standard and short stem groups, respectively (Tables 2 and 3, and Fig. 2). At 5 years there were no differences in THA (DDH: 96.7%, number 1 short stem 97.5%, SRS 97.3%, standard 98.6%) or stem only (DDH: 97.6%, number 1 short stem 99.0%, SRS 97.3%, standard 98.2%) survival between the different short stems options or when compared to the standard group (Tables 4 and 5 and Fig. 3). Ten-year comparison between the short stem groups was limited due to the fact that only a smaller number of patients were followed up for a longer time and there were no patients in the number 1 short stem group (as only available for 2015).

**Table 2:** All Cause Survival (THA) for the standard and short Exeter^**®**^ stem groups

**Time (Years)**	**Standard Exeter, *n* = 698**				**All Short Stem Variant Exeter, *n* = 665**				**Log Rank** ***P*-value**
	**No. Risk**	**Events**	**Unadjusted** **Survival (95% CI)**	**Adjusted**^**a**^ **Survival (95% CI)**	**No. Risk**	**Events**	**Unadjusted** **Survival (95% CI)**	**Adjusted**^**a**^ **Survival (95% CI)**	**Adjusted**
1	685	5	99.1% (98.5–99.8)	99.3% (98.8–99.9)	650	10	98.5% (97.6–99.4)	98.1% (96.9–99.3)	0.26
2	669	4	98.6% (97.7–99.4)	98.6% (97.7–99.5)	612	6	97.6% (96.4–98.7)	97.2% (95.8–98.6)	
5	634	3	98.6% (97.1–99.1)	98.3% (97.3–99.3)	352	2	97.2% (95.9–98.5)	96.9% (95.4–98.4)	
10	529	8	96.7% (95.4–98.1)	97.0% (95.7–98.4)	42	3	96.2% (94.5–97.9)	96.0% (94.3–97.8)	

^a^Adjusted to sex, age group and ASA

**Table 3:** Stem-only survival for the standard and short Exeter^**®**^ stem groups

**Time (Years)**	**Standard Exeter, *n* = 698**				**All Short Stem Variant Exeter, *n* = 665**				**Log Rank** ***P*-value**
	**No. Risk**	**Events**	**Unadjusted Survival (95% CI)**	**Adjusted**^**a**^ **Survival (95% CI)**	**No. Risk**	**Events**	**Unadjusted** **Survival (95% CI)**	**Adjusted**^**a**^ **Survival (95% CI)**	**Adjusted**
1	686	4	99.3% (98.7–99.9)	99.5% (99.0–99.9)	653	8	98.8% (98.0–99.6)	98.5% (97.4–99.6)	0.35
2	670	4	98.7% (97.9–99.5)	98.7% (97.9–99.6)	618	3	98.3% (97.4–99.3)	98.1% (96.9–99.3)	
5	635	3	98.2% (97.3–99.2)	98.7% (97.4–99.4)	358	2	98.0% (96.8–99.1)	97.8% (96.5–99.0)	
10	532	4	97.5% (96.4–98.7)	97.8% (96.6–98.9)	42	3	96.9% (95.3–98.5)	96.9% (95.2–98.5)	

^a^Adjusted to sex, age group and ASA

**Table 4:** All-cause unadjusted survival (THA) for the standard and short Exeter^**®**^ stem subgroups

**Time (Years)**	**Standard Exeter, *n* = 698**			**DDH Exeter, *n* = 229**			**No.1 Short Stem Exeter, *n* = 208**			**Short Revision Stem, *n* = 227**		
	**No. Risk**	**Events**	**Unadjusted** **Survival** **(95% CI)**	**No. Risk**	**Events**	**Unadjusted** **Survival** **(95% CI)**	**No. Risk**	**Events**	**Unadjusted** **Survival** **(95% CI)**	**No. Risk**	**Events**	**Unadjusted** **Survival** **(95% CI)**
1	685	5	99.1% (98.5–99.8)	224	3	98.7% (97.2–100.0)	204	3	98.6% (96.9-100.0)	222	4	98.2% (96.5–100.0)
2	669	4	98.6% (97.7–99.4)	207	3	97.3% (95.2–99.5)	192	2	97.5% (95.4-99.7)	213	1	97.8% (95.9–99.7)
5	634	3	98.6% (97.1–99.1)	146	1	96.7% (94.4–99.2)	43	0	97.5% (95.4-99.7)	163	1	97.3% (95.2–99.5)
10	529	8	96.7% (95.4–98.1)	18	2	95.2% (92.0–98.4)				24	1	96.7% (94.4–99.2)

*DDH* Developmental dysplasia of hip

**Table 5:** Stem only unadjusted survival for the standard and short Exeter^**®**^ stem subgroups

**Time (Years)**	**Standard Exeter, *n* = 698**			**DDH Exeter, *n* = 229**			**No.1 Short Stem Exeter, *n* = 208**			**Short Revision Stem, *n* = 227**		
	**No. Risk**	**Events**	**Unadjusted** **Survival** **(95% CI)**	**No. Risk**	**Events**	**Unadjusted** **Survival** **(95% CI)**	**No. Risk**	**Events**	**Unadjusted** **Survival** **(95% CI)**	**No. Risk**	**Events**	**Unadjusted** **Survival** **(95% CI)**
1	686	4	99.3% (98.7–99.9)	225	2	99.1% (97.9–100.0)	205	2	99.0% (97.7–100.0)	222	4	98.2% (96.5–100.0)
2	670	4	98.7% (97.9–99.5)	209	2	98.2% (96.5–100.0)	195	0	99.0% (97.7–100.0)	213	1	97.8% (95.9–99.7)
5	635	3	98.2% (97.3–99.2)	148	1	97.6% (95.6–99.7)	46	0	99.0% (97.7–100.0)	163	1	97.3% (95.2–99.5)
10	532	4	97.5% (96.4–98.7)	18	2	96.1% (93.1–99.1)				24	1	96.7% (94.2–99.2)

*DDH* Developmental dysplasia of hip

## Discussion

This study demonstrated that the Exeter^**®**^ short stem offers equivocal THA and stem-specific survival at 5- to 10-year follow-up. There were significant demographic differences between the standard and short stem groups, with the latter being more likely to be female and younger in age. However, this sex difference was only observed for the DDH and SRS groups, whereas the more recently introduced number 1 short stems for implants with a 37.5 mm or greater offset were more likely to be used in males.

The major limitations of this study were the relatively short follow-up time, no radiographic assessment for signs of loosening or stem alignment, no patient-reported outcome measures, the retrospective design and no standardized criteria for the use of the short stems. The major reason for the short follow-up was due to the fact that the implant data collection commenced at the study center in 2011 but the number 1 short stem for 37.5 mm offset and greater was only available from 2015. Although this study presented a relatively short follow-up, it is, to the authors’ knowledge, one of the longest follow-ups for non-DDH stems used for primary THA. The study center does not routinely follow up on all patients radiographically and therefore the current study did not report any radiographic assessment, which is a limitation. However, when the patients’ notes were assessed to record revision/mortality status, there was no documentation of pending cases with loose/failing stems. Furthermore, the postoperative radiographs were not assessed for alignment of stem height. Varus alignment is more likely with a short stem component [8], which is associated with an increased risk of implant failure but this was observed in older cemented THA designs such as the Charnley monoblock stem [16–18]. No patient-reported outcomes were assessed between the groups, as it was felt that these would not be clinically significantly different [8], but this might not be the case if there was a failure to achieve restoration of offset due to stem design [19]. The retrospective design is another limitation, especially in view of the observed differences in the patient demographics not only between standard and short stems but also among the three short stem groups. Although adjusted analysis was undertaken to account for this, there will likely be additional factors out with the control of the study such as proximal femoral dysplasia which is associated with DDH and may be more likely to be present in the short stem group [20]. There were no strict criteria for the use of the short stems and this was left to the surgeon’s discretion. A standard stem was used unless the anatomy of the proximal femur did not allow for the planned placement of trial broach, then a short stem was used, which may be more common in Dorr type A proximal femurs [3]. This may have had implications on the survivorship for the short and standard stems due to differences in the proximal femoral bone stock, but to the author’s knowledge, there is no published literature relating to this when using a cemented stem.

Most of the current evidence for short femoral stems is related to uncemented fixation, with no difference in survivorship but is associated with improved proximal femoral remodelling and less associated thigh pain [5, 21]. Lidder et al. [22] performed a systematic review assessing short uncemented metaphyseal loading stems and compared them with standard stems and demonstrated no differences in radiological outcomes or midterm survivorship. In contrast, there is limited literature reporting the survival of short cemented stems, more specifically, the Exeter^**®**^ V40 stem. The Exeter^**®**^ V40 stem is one of the most common stems employed for THA in the National Joint Registry (NJR), but the registry annual reports groups all Exeter^**®**^ V40 stem designs together (standard and short stems) when reporting revision risk [23]. For the acetabula combinations used in the current study of either a Contemporary cemented cup or a Trident uncemented shell, the NJR reports a 5-year revision risk of 1.4%, which is similar to the 5-year survival rate of 98.6% for the standard group reported in this study. Although, no significant difference was demonstrated in survival between short and standard Exeter^**®**^ V40 stems in the current study at 5 years, the survival was, however, this was approximately 1% lower for the short stem group for both THA and stem-only survivals.

Wyatt et al. [11], by using data from the New Zealand National Joint Registry, compared the survival of the Exeter^**®**^ short stem with that of “standard” Exeter^**®**^ stems. Similar to the current study, they separated DDH stems (offset of 35.5 mm or less) and number 1 short stems (37.5 mm and greater and 125 mm in length) and reported separate survivorship figures. They demonstrated a greater revision risk for the DDH stem group (hazard ratio 1.49, *P* = 0.001), compared to the standard stem group, whereas they found no difference between the number 1 short stem and standard stem groups (*P* = 476). In their DDH group, 1,501 THA were assessed, of which 98 received revisions, but only 18 did so due to femoral stem loosening, against 39 (42.4%) who received revision due to loosening of the acetabular component. This may suggest that the higher revision risk associated with their DDH group could, in part, be due to acetabular failure rather than being related specifically to the DDH short stem design. They also identified 657 THAs that underwent/used a number 1 short stem (125 mm) with a 37.5 mm or greater offset and found no difference in survival compared to the standard stem at a mean follow-up of one year with no stem failures reported. Martin et al. [12] assessed the survival of the number 1 short stem (37.5 mm or greater offset) Exeter^**®**^ for up to 3 years in 60 patients undergoing primary THA and reported that no stem had been revised or failed. The current study supports the findings of both studies and affirms the equivocal survival at a longer follow-up of 5- years.

To the authors’ knowledge, there was only one report regarding the use of the SRS (44/00/125) as a part of primary THA. By using data from the NJR, they identified 2,158 primary THA employing this implant and found a revision risk that was nearly double that observed with the standard Exeter^**®**^ stem at 10-years follow-up [10]. Periprosthetic fracture was one of the largest contributors to the revision of the SRS stem [10], which is consistent with the increased risk associated with the collarless polished taper slip design [24, 25]. A systematic review by Thompson et al. [24] found 25 reported cases of prosthetic fractures of Exeter^**®**^ stems and demonstrated that short (125 mm) stem length was more likely to fracture, and of the seven reported short stems, four were SRS (44/00/125). The SRS was designed for the cement-in-cement revision field [2]. However, the smaller stem geometry may explain the increased risk of implant fracture and may be potentially one of the reasons for the higher revision risk associated with this stem when used in primary THA [10]. However, there were no reported SRS fractures in the current series and the survival was equal to that observed with a standard stem. There is also an associated increased risk of periprosthetic fracture with shorter stem length around a cemented polished taper stem implant, such as the Exeter^**®**^ stem [25, 26]. Therefore the short-cemented Exeter^**®**^ stem may be subject to periprosthetic fracture, but the rate in the current series was low.

In conclusion, the Exeter^**®**^ short stems offer equivocal survival when compared to the standard stem at 5- to 10-year follow-up, which does not seem to be influenced by the short stem design. However, the follow-up was short, especially for the number 1 short stems (37.5, 44 and 50 offset) due to their more recent introduction and a longer-term follow-up is required. Patient demographics seem to influence the indication for the short stem and these should be accounted for in future comparative studies with the established standard Exeter^**®**^ stem.

## Data Availability

The data and materials of this study can be attained by contacting the corresponding author.
